# Retention Forces of Prosthetic Clasps over a Simulated Wearing Period of Six Years In-Vitro: Direct Metal Laser Melting Versus Dental Casting

**DOI:** 10.3390/ma13235339

**Published:** 2020-11-25

**Authors:** Moritz Mutschler, Florian Schweitzer, Sebastian Spintzyk, Jürgen Geis-Gerstorfer, Fabian Huettig

**Affiliations:** 1 Department of Dental Technology, Vocational School “Fritz-Ruoff”, Albert-Schäffle-Straße 7, D-72622 Nürtingen, Germany; moritz.mutschler@frs-nt.de; 2 Department of Prosthodontics, University Hospital Tuebingen, Calwerstr. 7/7, D-72076 Tübingen, Germany; Florian.Schweitzer@med.uni-tuebingen.de (F.S.); Fabian.Huettig@med.uni-tuebingen.de (F.H.); 3 Section “Medical Material Science & Technology”, University Hospital Tuebingen, Osianderstr. 2-8, 72076 Tübingen, Germany; geis-gerstorfer@mwt-tuebingen.de

**Keywords:** additive manufacturing, dental prosthesis, longevity, non-precious alloy, ageing

## Abstract

This study determinates the persistence of retention force in Akers-clasps for removable partial dentures made from Co-Cr alloy. Therefore, standardized computer-aided designed (CAD) clasp #1 specimens were made by direct metal laser melting (DMLM, n = 10) and by lost-wax dental casting (DC) of computer-aided manufactured (CAM) replicas (n = 10, DC) from two comparable Co-Cr alloys. The retention force was tested after manufacturing for 9000 cycles of setting and removal from a molar tooth crown analog made from zirconia; simulating in-vitro a duration of six years in service. The first and last 360 cycles (T0 and T1, 3 months each) of all specimens were selected for comparison of retention forces between the materials. A constant decrease of 6% from the initial retention force (T0 = 4.86 N, SD = 0.077; T1 = 4.57 N, SD = 0.037) was detected at the DC specimens, and an increase of 4% in DMLM specimens (T0 = 5.69 N, SD = 0.078; T1 = 5.92 N, SD = 0.077); all differences were statistically significant (*p* < 0.0001). Even if these deviations are not of clinical relevance, further studies and applications should investigate the fatigue behavior of laser melted Co-Cr-alloys for dental application.

## 1. Introduction

According to the standard DIN EN ISO 17296, generative manufacturing, also known as “3D printing” or “additive manufacturing” (AM), is subdivided into deposition and binding approaches.

The “deposition approach” means a thermosensitive material is heated or even melted and laid out in defined paths. After loss of thermal energy (cooling), the laid-out form remains. In the “binding approach”, the material is available as an unstructured layer and is then selectively melted and bonded by induced energy.

Processes relevant for the dental industry that are assigned to the binding process are [1]:

- Stereolithography
- Direct Light Processing (DLP)
- Direct Metal Laser Melting (DMLM)

DMLM allows the additive production of metals. This process is known under brand names such as Direct Metal Laser Sintering (DMLS, Fa. EOS GmbH, Kailingen, Germany), LaserCUSING (Fa. Concept Laser GmbH, Lichtenfels, Germany) or Direct Metal Laser Melting (DMLM, Fa. GE Additive, Garching/Munich, Germany). These are all partly protected designations, but generally they describe the same process [2]. The term “Direct Metal Laser Melting” (DMLM) is used in the following.

DMLM is performed in a chamber containing a heatable building platform that can be moved in vertical direction to allow the layering. This platform is tempered from 80 °C to 200 °C to avoid distortions. A metal alloy powder (particle size of 25–45 µm) is applied to this platform [3].Tool steel or stainless steel, cobalt-based alloys, aluminum alloys, gold zinc, titanium or even magnesium can be used for this purpose [2].

A laser beam, guided by mirrors, “travels” over the previously defined areas and thereby selectively melts the metal powder completely: adjacent particles melt and form the defined structure of the layer. Thereafter, the building platform is lowered by dimension of one layer thickness and a new layer of the powder alloy is applied. The following layer is melted by laser beam and merges the particles with the underlying, last layer at the same moment.

DMLM is predominantly found in the aerospace and engineering industry, but also established in medical technology and the dental industry [4,5].

Generally, for dentistry or rather dental technology, DMLM of alloys allows us to overcome conventional lost-wax casting of metals as well as computer-aided design/ computer-aided manufacturing (CAD/CAM)-based subtractive fabrication of fixed or removable partial dentures, especially when it comes to complex designs such as combined solutions with patrices and matrices (such as telescopic crowns) or even one-piece casted clasp-retained removable partial dentures (RPDs).

Aside from financial efforts, a skepticism towards digitized prosthesis fabrication touches upon non-inferiority of “novel” approaches regarding dimensional accuracy (clinical fit) as well as material behavior (clinical long-term performance) to trust in the cost-benefit of solutions such as DMLM.

The present study was conducted in order to compare a DMLM procedure based on a digital workflow with a conventional dental casting of clasps, both from a non-precious cobalt-chromium alloy for RPDs. Therefore, the study addresses the retention forces of clasps delivered from both fabrication methods, before and after a simulated wearing period of six years.

The following two null hypotheses were set:

1. There is no statistically significant difference in the retention forces between the DMLM-manufactured CoCr-alloy clasps (Remanium star CL, Dentaurum GmbH & Co. KG, Ispringen, Germany) and the casted CoCr-alloy clasps (Remanium GM 800+, Dentaurum GmbH & Co. KG, Ispringen, Germany).

2. There is no statistically significant difference in the retention force before and after a simulated wearing period of six years for the DMLM-fabricated and cast clasps.

## 2. Materials and Methods

### 2.1. Fabrication of Clasp Bearing Tooth Analogs

**Figure 1:**
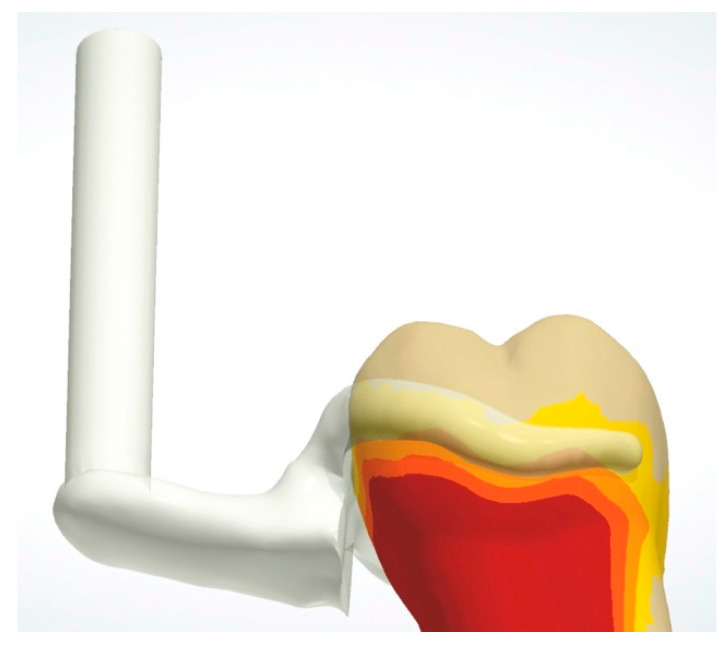
Design of the Asker clasp at the digitized tooth crown with a prepared rest area. The clasp was provided with a fixture to connect it to testing device.

A left maxillary first molar (FDI 26) resin training tooth (Frasaco GmbH, Tettnang, Germany) was chosen as reference and a mesial occlusal rest for an Asker clasp was prepared with a ball-shaped yellow-ringed rotary instrument of 2.5 mm in diameter (FG Ho-001 F 025, Horico Comp., Berlin, Germany, see Figure 1). The tooth was digitized by means of a desktop scanner (D2000, 3Shape, Copenhagen, Denmark) and is available as an STL-dataset (supplementary files).

A total of 20 clasp bearing tooth analogs were made from Zirconia (DD Bio ZW, ISO color shade 1000, Dental Direkt GmbH, Spenge, Germany; LOT: 6331412002) applying a standard CAD/CAM unit (CORiTEC 350i Loader (Imes-icore GmbH, Eiterfeld, Germany). These zirconia replicas were sintered at 1450 °C for 16 h (LHT 02/17 LB Speed, Nabertherm GmbH, Lilienthal/Bremen, Germany), as instructed by the manufacturer.

### 2.2. Fabrication of Asker Clasps Test Specimens

A circumferential clasp with occlusal support (Asker clasp or clasp #1; E-shaped) [6] with a maximum undercut of 0.25 mm was designed on the CAD tooth dataset (Software Dental Designer Vers. 16.4.0; 3Shape, Copenhagen, Denmark) of the crown analog (Figure 1, STL in supplementary files). A fixture was digitally attached to the clasp-design (Meshmixer Vers. 3.4.35, Autodesk Corp., San Rafael, CA, USA) in order to later fix the clasp within the testing device. This fixture was positioned in accordance with the insertion direction of the claps on the tooth crown. A total of n = 20 clasp test specimens were manufactured as follows.

1. Fabrication of clasps by direct metal laser melting (DMLM): Ten test specimens of the final Asker clasp CAD data set were fabricated from the Co-Cr-alloy (Remanium star CL, Dentaurum GmbH & Co. KG, Ispringen, Germany) applying the Mlab Cusing Machine (Concept Laser, Lichtenfels, Germany).
2. Fabrication of clasps by dental casting (DC): Ten test specimens were milled based on the Asker clasp CAD data from a wax-like resin which allows to be burned away to nothing (StarWax blank grey, Dentaurum GmbH & Co. KG, REF 120-230-00 LOT 100633, Ispringen, Germany). Five specimen were invested to one muffle at a time (rema exakt, Dentaurum GmbH & Co. KG, Ispringen, Germany) and casted in lost-wax technique with the Co-Cr-alloy (remanium GM 800+, Dentaurum GmbH & Co. KG, Ispringen, Germany) in a high-frequency casting unit (Megapuls compact, Dentaurum GmbH & Co. KG, Ispringen, Germany) from pre-heated condition of 950 °C (MIHM-Vogt GmbH & Co KG, Stutensee, Germany).

The composition of the alloys for DMLM and DC are described in Table 1.

### 2.3. Testing Device, Test Setup, and Testing

**Figure 2:**
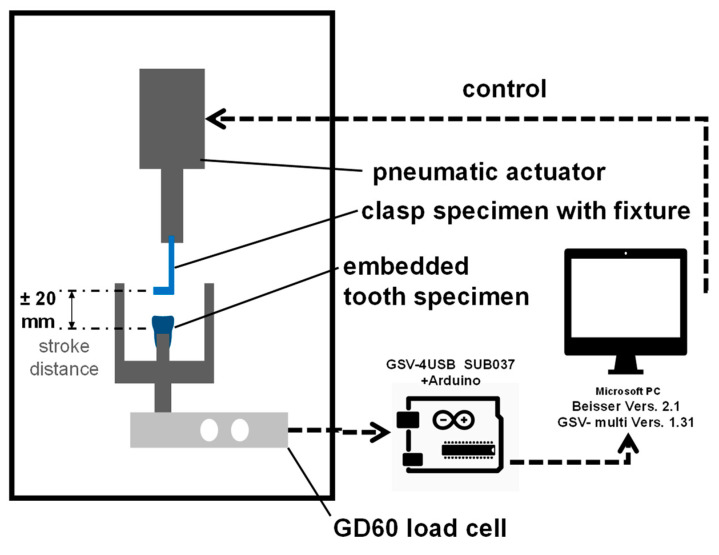
Schematic drawing of the testing device and control/ data acquisition units.

**Figure 3:**
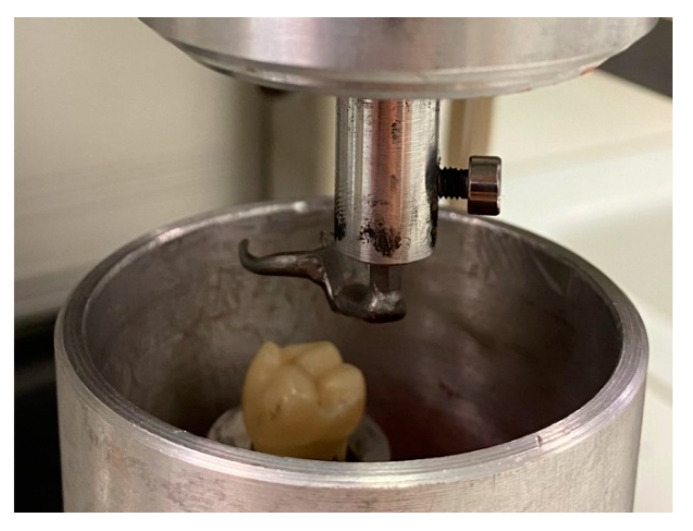
Test setup of a DC clasp in lifted position to the clasp bearing tooth crown analog (embedded in polymethylmethacrylate).

The construction of the pneumatic testing device (Mader Typ PMVH 5/2-1/8, Mader GmbH & Co KG, Leinfelden-Echterdingen, Germany) as well as the integration of the specimens for measurements while testing is shown in Figure 2 and Figure 3. The force measurement was performed by a double bending bar (KD60, ME Messsysteme GmbH, Hennigsdorf, Germany) of 100 N nominal load and 0.1% measuring accuracy connected to the hardware control (GSV-4USB Sub D37m ME Messsysteme GmbH, Hennigsdorf, Germany). The control and recording of the measurements were carried out by software (Biter 2.1 and GVCmulti 1.31, ME Messsysteme GmbH, Hennigsdorf, Germany).

A complete test cycle was defined as the movement from the settled clasp to a complete removal of the clasp and the resetting of the clasp to the tooth by the lift. The cycle of evaluation is defined as the half movement of the complete cycle: the removal of the clasp from the passive set at the tooth until the upmost lift position (+ 20 mm). Data acquisition was delivered by the software with 30–80 force measurements (in Newton) per evaluated cycle accompanied by the lift position in TDMS files (National Instruments, Austin, TX, USA). The testing speed was 40 cycles per minute (= 40 Hz).

Both specimen “clasp” and “tooth crown analog” were brought together to be clamped into the testing machine. Beforehand, the tooth crown analog was fixed into a holder using a self-polymerizing polymethylmethacrylate (Palavit G, Kulzer GmbH, Hanau, Germany) (Figure 3).

The passive set of the clasp on the tooth crown was validated by a displayed value of 0 N by the measuring software. Therewith, the clasp is aligned to the occlusal support on the tooth crown analog without force in neither occlusal nor apical direction of its insertion.

Thereafter, each test specimen experiences a number of 9000 cycles to simulate four insertions and removals of a prosthesis every 24 h, plus 60 d to allow data for full 6 years (= 4 × 365 d × 6 + 4 × 60 d). The test was carried out at room temperature under constant wetting of the testing side with distilled water.

### 2.4. Statistical Methods

The number of specimens was set to 10 per group in order to detect a significant difference of 4% with 99% power under assumption of an alpha of 5% and 1% standard deviation in force distribution.

Data analysis was performed with JMP statistical package (Version 15.1, SAS Institute GmbH, Heidelberg, Germany). Therefore, TDSM files were extracted to XLS (Version 15, National Instruments) and copied to JMP Tables.

The peak of the retention force measurements during each cycle of each specimen was identified by scripting with a time series analysis. The peaks of ten cycles of all specimens are grouped and averaged in order to depict data over time (0–900 “cycles * 10”).

The moment “T_0_” was defined as the set of single peaks from the first 360 cycles (0–360) of all specimens in a group (DC, DMLM), simulating the removal force of 3 months (90 days multiplied with 4 removals of the prosthesis per day).

The moment “T_1_” was defined as the set of single peaks from the last 360 cycles (8640–9000) of all specimens in a group (DC, DMLM), simulating the removal force at 6 years ± 1 month in service.

The distributions of retention forces of all specimens of each material (DMLM, DC) and at each point in time (T_0_, T_1_) were tested for normality using Sharpiro-Wilk test with an alpha = 0.05. In case of normal distributions, the means were pairwise tested with Students t-test, otherwise applying the Kruskar-Wallis rank sum test; both with an alpha = 0.05.

The analysis follows the null hypotheses that no difference in values is present between the materials as well as the points in time of testing. A relevant difference was expected to exceed 15% one-sided from the shared mean or 2 N.

## 3. Results

### 3.1. Retention Forces over Time

**Figure 4:**
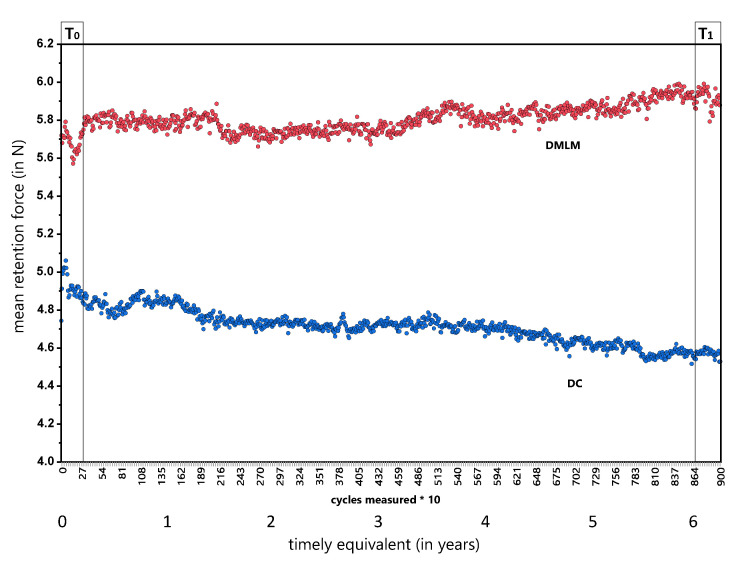
Mean retention forces of clasps over time. Each dot either of blue (DC) or red (DMLM) color, represents the 10-clasp specimen measured for 10 removal and resets on the artificial tooth crown analogs. The X-Axis shows the cycles and the timely equivalent in years (1 year =1460 cycles). Please be aware that for visual reasons the y-axis was stretched by cut of < 4.0 N.

Figure 4 illustrates the measured retention forces during 9000 cycles in sets of 10 for all specimens. There was a slight linear descending of mean retention forces for DC and a slight ascending for DMLM between T_0_ and T_1_, respectively.

### 3.2. Comparison of DC and DMLM at T_0_ and T_1_

**Figure 5:**
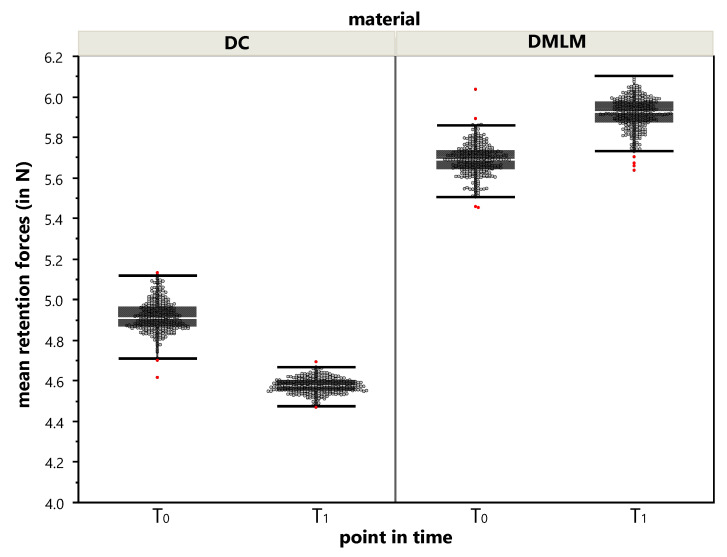
Distribution of mean retention forces during T_0_ and T_1_. A dot represents the mean retention force of the 10-clasp specimen from a single measurement out of the 320 cycles (removals and resets on the artificial tooth crown analog) during T_0_ and T_1_. Please be aware that, for visual reasons, the y-axis was stretched by a cut of <4.0 N. The superimposed boxplots give the median, quartiles, and whiskers to minimum and maximum; outliers are marked red.

Distribution of the mean retention forces were not normally distributed in the 10 specimens at T0 and T1 (360 cycles each) for DC as well as not for DMLM. The distributions can be found in Figure 5.

DC revealed a mean retention force of 4.86 N, SD = 0.077 at T_0_ and 4.57 N, SD = 0.037 at T_1_ with a statistically significant difference (*p* < 0.0001; Z = 21.89). The reduction of retention force in DC clasps is 6% from its initial value. 

DMLM revealed a mean retention force of 5.69 N, SD = 0.078 at T_0_ and 5.92 N, SD = 0.077 at T_1_ with a statistically significant difference (*p* < 0.0001; Z = −20.86). The retention force in DMLM clasps gains 4% from its initial value.

In consequence, the distributions of DC and DMLM retention forces are statistically significant different from each other (*p* < 0.0001) in both points in time. However, the difference neither exceeded 2 N, nor does the one-sided deviation from the shared mean (8.5% at T_0_ and 12.9% at T_1_) indicates a relevant difference.

## 4. Discussion

The test setup delivered reliable data with a scattering in scope of measurement uncertainty of 0.1% throughout all tests. No clasp experienced a fracture during testing.

Both the DC and the DMLM group remained below the maximum retention force of 10 N required in the literature. Forces above 10 N may cause damage to the periodontium. In general, a force of 3to 5 N [7] and up to 7.5 N [8] are recommended per clasp bearing tooth.

The difference of initial retention forces between DC and DMLM might be due to the manufacturing process of both the clasps and the zirconia tooth crown analog.

Both were examined with a qualitative post-hoc analysis by matching of the specific tooth crown analogs as well as the clasps, which derived minimum and maximum force values in both groups. The original STL data set was used as ground truth and compared by Geomagic Control X Software (Version, 3D Systems, Rocket Hill, SC, USA) with the scans derived by the D2000 stationary scanner (Amann Girrbach GmbH, Pforzheim, Germany).

Therefore, the clasps were powered with O-spray white (#230233, Scheftner Dental Alloys, Mainz, Germany) from 20 cm distance prior to scanning.

For the zirconia teeth, no exceeding of the range of nominal deviation of 50 µm was detected.

For the clasps, deviations of the retention clasp were higher than the reciprocal clasp in both groups, and ranged between 0.1 mm (reciprocal) and 0.3 mm (retention). Thus, in both groups the tips of the clasps were irreversibly bent up by this extent during testing in the range of the designed undercut (0.25 mm). In consequence, this implies that no abrasion or wear was present “tooth-side”, but has to be expected for enamel in the clinical situation. Therewith, the detected amount of bending might be less extensive because the irreversible deformation emerges at a later point in time than abrasion of enamel takes place. This should be clarified in future studies.

Since DMLM showed 20% higher retention forces than DC clasps of same design and rises in force apart from the findings mentioned before, this observation has to be taken into account and needs further scientific investigation.

A reasonable explanation for this finding might be the difference in material parameters due to the slightly different compositions of the alloys and their processing. Concretely, this equates to the amount of molybdenum in DC and tungsten in DMLM (see Table 1).

The DMLM fabricated non-precious alloy from this study was reported to have a significant higher technical elastic limit (Rp 0.2% = 783 MPa) than a comparable bonding alloy for dental casting (Rp 0.2% = 581 MPa) [9]. However, in the present study, the applied casting alloy is reported with Rp 0.2% = 720 MPa by the manufacturer and thereby comparable to the Rp 0.2% = 783 MPa of the same applied DMLM alloy. For both alloys, the elastic modulus is given by the manufacturer with 230,000 MPa. However, the DC and DMLM alloys differ towards tensile strength (960 MPa vs. 1150 MPa) and ultimate elongation (4% vs. 8–11%). This implies that the induction of minimal forces over time may have an impact on changes in the structure within one of the metal alloys, such as cold setting or hardening or embrittlement, as described in [10].

Consequently, the retention force can be influenced by an adapted design of the forearm of the clasp, accordingly: The retention force increases with increased thickness and cross-section of the forearm [11].

On the other hand, this difference can be a matter of “micro-misfits” which can arise from the manufacturing chain of the clasps in the DC group. The process of DC-group does not fully correspond to the working procedure used in practice. In order to be able to design reproducible clasp test specimens, the data set of the clasp test specimen was first created subtractively, embedded and finally manufactured by means of dental casting. This represents a “semi-digital workflow” and does not correspond to the procedure commonly used in practice, in which the object to be created is modelled manually in wax on a refractory model. A current alternative could be the additive manufacturing of resin clasps to be casted, as described in Torabi et al. [12].

However, clinically, such a deviation (in terms of misfit) might not be detectable to the dental practitioners because of visible gaps, for instance, while this was also not the case within this in-vitro setting.

Even if a statistically significant difference of the retention forces between T_0_ and T_1_ was detected in both groups, these differences are below the clinically relevant bandwidth mentioned before. Interestingly, the differences of retention forces are diverging (loss in DC and gain in DMLM). This can be attributed to the compositions as well as changes in microstructure of the alloys.

Mechanically, according to Wu et al. [13], a Co-Cr alloy processed by DMLM reaches higher yield strength (884.37; ± 8.96 MPa > 758.73; ± 30.94 MPa) than a casted alloy and can therefore be subjected to greater stress until plastic deformation occurs. This goes in hand with our findings of significantly higher tensile strength in DMLM (1307.50 ± 10.65 MPa) than in DC (758.73 ± 25.85 MPa) as well as with the findings of Zhou et al. [14].

The smaller proportion of the total void volume of the DMLM group was also observed by Schweiger et al. [10] in their study. The porosity that often occurs in traditional casting processes leads to a changed microstructure and thus to other mechanical properties that are less adjusted or controllable and can also increase the susceptibility to corrosion [3].

During the manufacturing process of the DMLM micron areas are melted, which cool down faster and thus lead to a finer-grained microstructure. Due to the Hall-Petch relationship, the strength of metallic materials is inversely proportional to the grain size, which is why the higher retention forces are found in the group DMLM.

All issues mentioned above are major limitations of the present study since a more in-depth insight towards metallurgy as well as the quantification of fit and wear of the produced clasps is lacking.

In daily practice, the DMLM procedure offers advantages over the dental casting. First, Koutsoukis et al. reported that the DMLM technique enables a faster and more cost-effective restoration without having to sacrifice quality [3]. Second, Xin et al. found that a Co-Cr alloy produced by DMLM has a lower ion release and better biocompatibility compared to the traditional casting process [15]. The findings of the present study show that more research is needed to establish valid digital workflows (e.g., design according to the manufacturing process and alloy) as well as the influence of the long-term behavior on biological and mechanical properties.

## 5. Conclusions

Even after a simulated wearing time of six years, both methods show a successful performance of retention within the scope of clinical application, even if the retention forces are statistically different from each other. The increased scattering of retention forces in the DMLM procedure has to be clarified in further studies in order to be able to guarantee reproducibility in the production of clasp-retained removable partial dentures and their long-term behavior.

In summary, it can be stated that the DMLM method seems to be equivalent to dental casting with regard to retention force.

## Figures and Tables

**Table 1:** Composition of the non-precious alloys under observation.

Element	DMLM (Remanium Star CL) % *w*/*w*	DC (Remanium GM 800+) % *w*/*w*
Co	60.5	58.3
Cr	28	32
W	9	1.5
Si	1.5	1
Mo	0	6.5
other	Mn, N, Nb Fe < 1	C, N < 1

## Supplementary Materials

The following are available online at https://www.mdpi.com/1996-1944/13/23/5339/s1, STL File of the clasp bearing tooth analog and the STL file of the fitting clasp.
